# Leveraging genetic correlations and multiple populations to improve genetic risk prediction for non-European populations

**DOI:** 10.21203/rs.3.rs-3741763/v1

**Published:** 2023-12-25

**Authors:** Leqi Xu, Geyu Zhou, Wei Jiang, Leying Guan, Hongyu Zhao

**Affiliations:** 1 Department of Biostatistics, Yale School of Public Health, New Haven, CT, USA.; 2 Program in Computational Biology and Bioinformatics, Yale University, New Haven, CT, USA.

**Keywords:** Bayesian high-dimensional regression, Multi-population genetic risk prediction, Cross-population genetic correlation, Continuous shrinkage prior

## Abstract

The disparity in genetic risk prediction accuracy between European and non-European individuals highlights a critical challenge in health inequality. To bridge this gap, we introduce JointPRS, a novel method that models multiple populations jointly to improve genetic risk predictions for non-European individuals. JointPRS has three key features. First, it encompasses all diverse populations to improve prediction accuracy, rather than relying solely on the target population with a singular auxiliary European group. Second, it autonomously estimates and leverages chromosome-wise cross-population genetic correlations to infer the effect sizes of genetic variants. Lastly, it provides an auto version that has comparable performance to the tuning version to accommodate the situation with no validation dataset. Through extensive simulations and real data applications to 22 quantitative traits and four binary traits in East Asian populations, nine quantitative traits and one binary trait in African populations, and four quantitative traits in South Asian populations, we demonstrate that JointPRS outperforms state-of-art methods, improving the prediction accuracy for both quantitative and binary traits in non-European populations.

## 1 Introduction

Polygenic Risk Scores (PRS), the weighted sum of risk alleles across a collection of genetic variants, have seen active development for predicting complex traits in recent years. PRS have demonstrated its ability to identify individuals with high disease risk, which can be applied for early disease prevention, tailored therapies, and personalized medicine [1–4]. However, most current PRS have been developed primarily for European populations, due to the dominance of large European cohorts in Genome-Wide Associations Studies (GWAS) [5]. Consequently, the prediction accuracy of PRS suffers for non-European populations [6].

Several factors contribute to the limited predictive efficacy of PRS in non-European populations. First, GWAS summary statistics for non-European populations are limited. Additionally, distinctive genetic structures exist between European and non-European populations, including different linkage disequilibrium (LD) patterns and unique casual SNPs. Furthermore, the number of SNPs also varies across populations. This gap in genetic risk prediction performance can exacerbate health disparities [6, 7], leading to an urgent need to improve the PRS prediction accuracy for non-European populations.

To address this need, there has been an increase in the number of GWAS focused on non-European populations [8–15], complemented by the developments of various models tailored for multi-population PRS predictions. These models employ different strategies to leverage multiple GWAS results, including utilizing multiple populations, assuming sparse distributions for genetic risk variants across populations, and accounting for genetic correlations among populations [16–22]. However, to our knowledge, there is no method that integrates all these components under a coherent framework in the absence of individual-level validation data —a common situation in real data applications. There is also a lack of investigation of the relative contributions of considering each of the three components in improving prediction accuracy. Moreover, there is a need for benchmarking recently developed multi-population PRS methods to guide future developments and applications of multi-population PRS methods.

In the present study, we introduce JointPRS, a Bayesian framework designed for simultaneously modelling GWAS summary statistics from multiple populations to enable cross-population predictions. JointPRS can automatically estimate heterogeneous SNP effects and provide chromosome-wise assessments of cross-population genetic correlations, and integrate them into PRS construction, even in the setting when only GWAS summary statistics are available during training. The efficacy of JointPRS is evaluated through extensive simulations and applications to real data, including 22 quantitative traits and four binary traits in East Asian (EAS) populations, nine quantitative traits and one binary trait in African (AFR) populations, and four quantitative traits in South Asian (SAS) populations. We illustrate the benefits of integrating genetic correlation structures and jointly using multiple populations over exclusive reliance on the target and the auxiliary European groups. In addition, we compare JointPRS with other methods, including PRS-CSx [17], MUSSEL [18], PROSPER [19], SDPRX [20], XPASS [21], and BridgePRS [22], with and without a validation dataset. Our results illustrate the distinct contributions of joint modelling and genetic correlations to prediction accuracy, and suggest JointPRS as a promising method for predicting complex traits across populations with the auto-tuned JointPRS achieving comparable performances as top-performing alternative approaches that require heavy tuning on individual-level validation sets.

## 2 Results

### 2.1 Overview of JointPRS

**Fig. 1:**
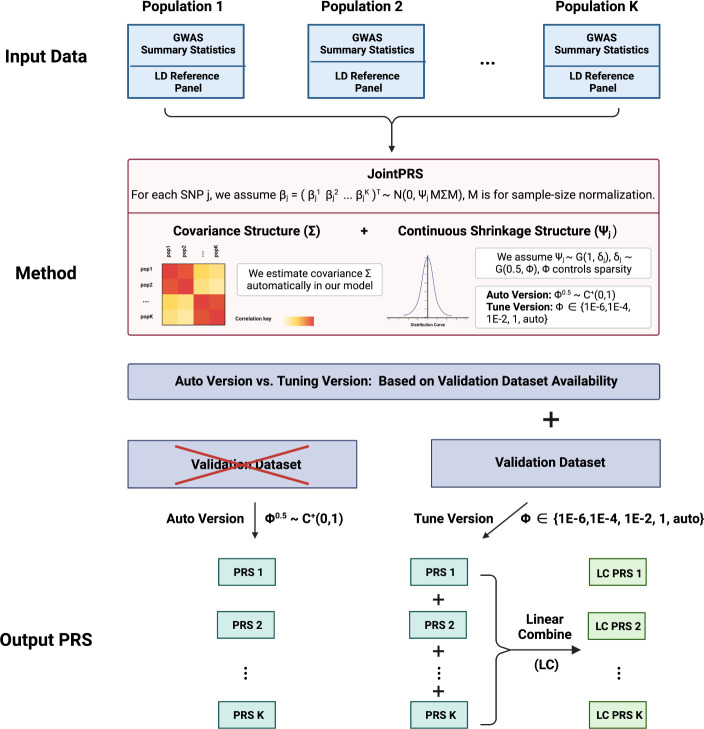
JointPRS Workflow. The pipeline of using JointPRS for PRS estimation under two scenarios: without and with a validation dataset.

JointPRS is based on a joint model for multiple populations under the Bayesian high-dimensional regression framework (Figure 1). The model has the following form

(1) y=Xβ+ϵ,

where

$$y=\left(\begin{array}{c} y_{N_{1}\times 1}^{1}\\ y_{N_{2}\times 1}^{2}\\ \vdots \\ y_{N_{K}\times 1}^{K} \end{array}\right),X=\left(\begin{array}{cccc} X_{N_{1}\times S}^{1} & 0 & \cdots & 0\\ 0 & X_{N_{2}\times S}^{2} & \cdots & 0\\ \vdots & \vdots & \ddots & \vdots \\ 0 & 0 & \cdots & X_{N_{K}\times S}^{K} \end{array}\right),\beta =\left(\begin{array}{c} \beta_{S\times 1}^{1}\\ \beta_{S\times 1}^{2}\\ \vdots \\ \beta_{S\times 1}^{K} \end{array}\right),\epsilon =\left(\begin{array}{c} \epsilon_{N_{1}\times 1}^{1}\\ \epsilon_{N_{2}\times 1}^{2}\\ \vdots \\ \epsilon_{N_{K}\times 1}^{K} \end{array}\right).$$

Here, K is the number of populations, $N_{k}$ denotes the sample size for population k, and S represents the number of SNPs that are available in at least one population. The vectors $y^{k},\;X^{k},\;\beta^{k}$ and $\epsilon^{k}$ correspond to the standardized phenotype vector, the column-standardized genotype matrix, the standardized effect size vector for SNPs, and residuals in population k respectively, with potential missing elements in the genotype matrix and effect size vector that we will discuss in detail in the following effect size model for each genetic variant.

When SNP j is available for all K populations, the effect size $\beta_{j}$ for SNP j across K populations is modeled with a correlated Gaussian prior:

(2) $$\beta_{j}={\left(\beta_{j}^{1}\beta_{j}^{2}\cdots \beta_{j}^{K}\right)}^{T}\;\sim \;N\left(0,\Psi_{j}M\Sigma M\right)$$

with

$$M=\mathrm{diag}\left(\frac{1}{\sqrt{N_{1}}},\frac{1}{\sqrt{N_{2}}},\cdots ,\frac{1}{\sqrt{N_{K}}}\right),\Sigma =\left(\begin{array}{cccc} \sigma_{1}^{2} & \sigma_{12} & \cdots & \sigma_{1K}\\ \sigma_{21} & \sigma_{2}^{2} & \cdots & \sigma_{2K}\\ \vdots & \vdots & \ddots & \vdots \\ \sigma_{K1} & \sigma_{K2} & \cdots & \sigma_{K}^{2} \end{array}\right).$$

Here, we can further illustrate the covariance matrix Σ as the transformation of the correlation matrix

$$\Sigma =\left(\begin{array}{cccc} \sqrt{\sigma_{1}^{2}} & 0 & \cdots & 0\\ 0 & \sqrt{\sigma_{2}^{2}} & \cdots & 0\\ \vdots & \vdots & \ddots & \vdots \\ 0 & 0 & \cdots & \sqrt{\sigma_{K}^{2}} \end{array}\right)\left(\begin{array}{cccc} 1 & \rho_{12} & \cdots & \rho_{1K}\\ \rho_{21} & 1 & \cdots & \rho_{2K}\\ \vdots & \vdots & \ddots & \vdots \\ \rho_{K1} & \rho_{K2} & \cdots & 1 \end{array}\right)\left(\begin{array}{cccc} \sqrt{\sigma_{1}^{2}} & 0 & \cdots & 0\\ 0 & \sqrt{\sigma_{2}^{2}} & \cdots & 0\\ \vdots & \vdots & \ddots & \vdots \\ 0 & 0 & \cdots & \sqrt{\sigma_{K}^{2}} \end{array}\right).$$

Here $\rho_{k_{1}k_{2}}$ denotes the cross-population genetic correlation between population $k_{1}$ and $k_{2}$. In addition, $\Psi_{j}$ is the sample-size normalized effect size for SNP j shared across K populations. This shared effect $\Psi_{j}$ is modeled by a continuous shrinkage prior following the PRS-CSx model and assumed to follow the gamma distribution $\Psi_{j}\;\sim \;G\left(1,\delta_{j}\right),\;\delta_{j}\;\sim \;G\left(\frac{1}{2},\phi \right)$ [17]. Here, we assume ϕ follows a standard half-Cauchy prior $\phi^{\frac{1}{2}}\;\sim \;C^{+}(0,\;1)$ for JointPRS-auto when there is no validation dataset or consider it as a tuning parameter using set {1E − 6, 1E − 4, 1E − 2, 1, *auto*} when there exists a validation dataset.

For SNP j available only in a subset of populations, this model is truncated to include only populations that contain SNP j.

Therefore, we can use the following index matrix T to encode the SNP missing patterns across the whole genome for different populations:

$$T=\left(\begin{array}{c} T_{1}{\;}^{T}\\ T_{2}{\;}^{T}\\ \vdots \\ T_{S}{\;}^{T} \end{array}\right)=\left(\begin{array}{cccc} T_{1}^{1} & T_{1}^{2} & \cdots & T_{1}^{K}\\ T_{2}^{1} & T_{2}^{2} & \cdots & T_{2}^{K}\\ \vdots & \vdots & \ddots & \vdots \\ T_{S}^{1} & T_{S}^{2} & \cdots & T_{S}^{K} \end{array}\right),T_{j}^{k}=\left\{\begin{array}{ll} 1 & SNP\;j\;\text{is}\;\text{available}\;\text{in}\;\text{population}\;k,\\ 0 & SNP\;j\;\text{is}\;\text{not}\;\text{available}\;\text{in}\;\text{population}\;k. \end{array}\right.$$

And each SNP j is assumed to follow a $sum\left(T_{j}\right)$-dimension multivariate correlated Gaussian model, with the truncated covariance matrix keeping only rows and columns corresponding to $T_{j}^{k}=1$ for k=1,…,K.

The residual component $\epsilon^{k}$ is assumed to follow a Gaussian distribution, denoted by $\epsilon^{k}\;\sim \;N\left(0,\sigma_{k}^{2}I_{N_{k}}\right)$. The variance component $\sigma_{k}^{2}$ here is assumed to follow a non-informative Jeffreys prior with its density distribution expressed as $f\left(\sigma_{k}^{2}\right)\propto \sigma_{k}^{-2}$. We note that the $\sigma_{k}^{2}$ are also the diagonal elements of the covariance matrix, thus avoiding the convergence issue. Since different populations do not share samples, we can assume the independence of $\epsilon^{k}$ across populations. Moreover, all upper triangle elements in the correlation matrix: $\rho_{12},\rho_{13},\cdots ,\rho_{K-1K}$ are assumed to follow a uniform distribution Uniform(0,1) and the correlation matrix is symmetric.

We note that the major difference between the JointPRS model and the PRS-CSx model [17] is that, PRS-CSx assumes the cross-population genetic correlation terms $\rho_{12},\rho_{13},\cdots ,\rho_{K-1K}$ equal zero so that the covariance matrix in the model will be a diagonal matrix, while JointPRS estimates the genetic correlation terms automatically from the training GWAS summary statistics.

### 2.2 Existing Methods

In this study, we compared the prediction accuracy of JointPRS with six existing methods for cross-population predictions: PRS-CSx [17], MUSSEL [18], PROSPER [19], SDPRX [20], XPASS [21], and BridgePRS [22]. These methods can be classified into two groups: multiple population models (including JointPRS, PRS-CSx, MUSSEL, and PROSPER) and two population models (including SDPRX, XPASS, and BridgePRS). For the multiple population models, all the available populations are jointly considered when estimating the PRS of a trait for a given population, and we denote these methods as “method_max” (“max” indicates that “maximum” number of populations are considered). In contrast, the two population models consider the European and the respective target population for each trait, and we denote these methods as “method_2” (“2” indicates that only European and the targeted populations are considered).

Some methods need a validation cohort to select model parameters. In scenarios where a validation dataset is unavailable, we only considered the automated versions of JointPRS and PRS-CSx (i.e., JointPRS_auto_max, PRScsx_auto_max), along with two other automated methods: SDPRX_auto_2 and XPASS_auto_2 (“auto” indicates no tuning parameters). In the presence of the validation dataset, we first evaluated the prediction accuracy using the optimally-tuned parameter for each population using four “best” methods: JointPRS_best_max, PRScsx_best_max, MUSSEL_best_max, and PROSPER_best_max (“best” indicates being optimally-tuned). SDPRX_auto_2 and XPASS_auto_2 were also considered in this comparison as they do not require tuning parameters, and the “auto” version will be their “best” version. As for BridgePRS, we found selecting either their joint model or their population-specific model with the optimal parameter is not predictive in some traits, so we linearly combined the PRS from two models suggested in their paper [22] and use this BridgePRS_linear_2 even in this “best” situation for comparison. We also compared JointPRS_linear_max, PRScsx_linear_max, MUSSEL_super_max, PROSPER_super_max, SDPRX_linear_2, XPASS_linear_2, and BridgePRS_linear_2. As suggested by PRS-CSx [17], both JointPRS_linear_max and PRScsx_linear_max linearly combined the standardized PRS from different populations. This combination is based on a validation dataset, using the same global shrinkage parameter. The optimal global shrinkage parameter is then chosen from the range {1E-6,1E-4,1E-2,1,auto} based on the same validation dataset to derive the final score. Similarly, SDPRX_linear_2 and XPASS_linear_2 linearly combined the standardized PRS of two populations to obtain the final PRS. Conversely, MUSSEL_super_max and PROSPER_super_max consider a super learning step that substitutes the linear combination by the non-linear machine learning techniques, and use the ensemble learning instead of selecting the optimally-tuned parameters, following their original work [18, 19]. In addition, as we mentioned above, BridgePRS_linear_2 linearly combined the PRS from the population-specific model with the PRS from the joint model using the optimal parameter as suggested by the authors [22].

All the multi-population PRS methods discussed above are organized in Table 1.

### 2.3 Simulations

We first compared the performance of JointPRS to three other existing methods PRS-CSx, SDPRX, and XPASS due to the reference panel availability under different simulation settings across populations. The simulated individual-level genotypes dataset which consists of 30,000 SNPs in total is based on the 1000 Genomes Phase 3 haplotype generated by HAPGEN2 [23]. The detailed simulation procedure is summarized in the Supplementary Notes (Individual-level Genotype Simulation Section). Finally, we obtained 40,000 individuals for the European population (EUR), 20,000 individuals for the East Asian population (EAS), and 10,000 individuals for the African population (AFR) in the training dataset. Moreover, for each population, we simulated validation and testing datasets, each consisting of 5,000 individuals. Here the validation dataset is used to estimate the LD matrix and tune parameters for methods we considered.

Then we generated the simulated effect sizes of each SNP j across the three populations under the following spike and slab model:

$$\left(\begin{array}{l} \beta_{EUR,j}\\ \beta_{EAS,j}\\ \beta_{AFR,j} \end{array}\right)\;\sim \;(1-p)\cdot \left(\begin{array}{l} \delta_{0}\\ \delta_{0}\\ \delta_{0} \end{array}\right)+p\cdot N\left(\left(\begin{array}{l} 0\\ 0\\ 0 \end{array}\right),\frac{1}{p\cdot S}\left(\begin{array}{ccc} \sqrt{h_{1}^{2}} & 0 & 0\\ 0 & \sqrt{h_{2}^{2}} & 0\\ 0 & 0 & \sqrt{h_{3}^{2}} \end{array}\right)\left(\begin{array}{ccc} 1 & \rho_{12} & \rho_{13}\\ \rho_{12} & 1 & \rho_{23}\\ \rho_{13} & \rho_{23} & 1 \end{array}\right)\left(\begin{array}{ccc} \sqrt{h_{1}^{2}} & 0 & 0\\ 0 & \sqrt{h_{2}^{2}} & 0\\ 0 & 0 & \sqrt{h_{3}^{2}} \end{array}\right)\right).$$

We considered the parameter values $h_{1}^{2}=h_{2}^{2}=h_{3}^{2}=0.3,\;\rho_{12}=0.6,\;\rho_{23}=0.8,\;\rho_{13}=0.5$, and S=30000. We examined three scenarios with different proportions of causal genetic variants: (1) p=0.005, (2) p=0.05, and (3) p=0.5. Based on these parameters and effect sizes, we utilized GCTA-sim [24] to generate phenotypes analyzed on the training, validation and testing dataset. PLINK2 [25] was further used to derive summary statistics in the training dataset.

**Fig. 2:**
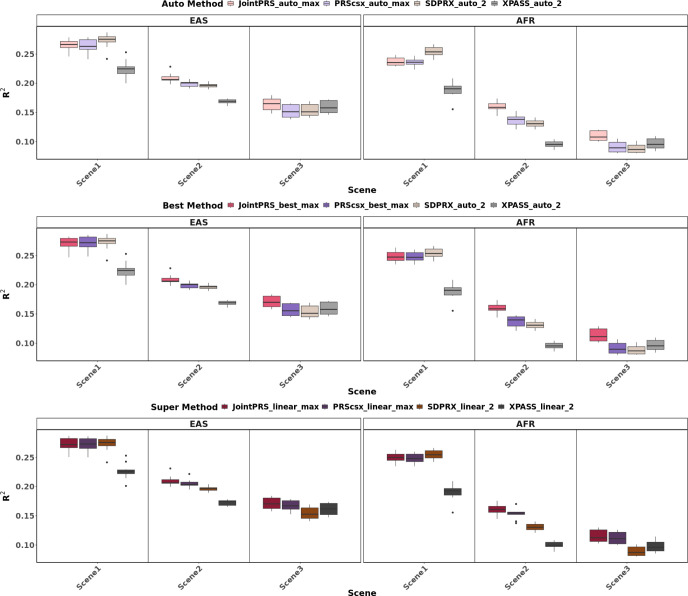
Method Comparison under Different Simulation Settings. The proportion of casual SNPs was set to be 0.005 (scenario 1), 0.05 (scenario 2), and 0.5 (scenario 3). Simulation in each scenario was repeated for 10 times. For each boxplot, the center line is the median and the lower and upper edges represent the 25^th^ and 75^th^ percentiles, and dark points are outliers.

We investigated the prediction accuracy of JointPRS, PRS-CSx [17], SDPRX [20] and XPASS [21] with and without the validation dataset. Figure 2 and Table S1 indicate that, generally, the tuned PRS (method_best and method_linear) of the four methods (JointPRS, PRScsx, SDPRX, and XPASS) had slightly better prediction accuracy than their corresponding auto results in our simulations. As the proportion of casual SNPs increased, the prediction accuracy decreased due to the increased number of causal SNPs and their reduced effect sizes, making the prediction task more challenging. We also note that both the tuned and auto versions of JointPRS outperformed other methods in most cases for the simulated EAS and AFR populations, suggesting that incorporating genetic correlation structure and integrating multiple populations can improve prediction accuracy among underrepresented populations.

### 2.4 UK Biobank Analysis

#### 2.4.1 Data preparation

We collected GWAS summary statistics from various consortia, including GLGC, GIANT, BBJ, PAGE, ICBP, BCX, UKB, DIAGRAM, BCAC, CARDIoGRAM, TRICL-ILCCO and LC3 [8–15, 26–34]. We removed duplicated SNPs and performed quality control following the LDHub guidelines, using the LDSC software [35, 36]. Additionally, we restricted the SNP list for each population to SNPs available in reference panels across all evaluated methods to ensure a fair comparison. For all methods, we used the 1000 Genomes Project as the reference panel.

In our evaluation analysis for the UK Biobank dataset, we first classified UK Biobank individuals into one of five super-populations from the 1000 Genomes Project following the procedure in SDPRX [20]: European (EUR), East Asian (EAS), African (AFR), South Asian (SAS), and Admixed American (AMR). The numbers of subjects in these populations are: 311,601 EUR, 2,091 EAS, 6,829 AFR, 7,857 SAS, and 636 AMR.

We obtained quantitative phenotypes of the UK Biobank subjects from their corresponding data fields. Notably, for systolic and diastolic blood pressures (SBP and DBP), we integrated both manual and automated readings, in line with the recommendations of the pertinent GWAS paper [28]. When analyzing LDL-cholesterol (LDL), Total cholesterol (TC), SBP and DBP, we excluded individuals lacking medication information (data fields 6177, 6153) and adjusted the remaining data following the guidelines from the relevant GWAS literature [8, 28]. For binary traits, we determined the cases and controls for participants in the UK Biobank based on the corresponding ICD-9, ICD-10, operation code and self-report code of the disease. We only considered females for breast cancer, and for all binary traits, the effective sample size was calculated as $\frac{4*N_{\text{case}}*N_{\text{control}}}{\left(N_{\text{case}}+N_{\text{control}}\right)}$, where $N_{\text{case}}$ and $N_{\text{control}}$ denote the number of cases and controls, respectively.

We analyzed a total of 22 quantitative traits, evaluating the prediction accuracy of the derived PRS for each trait separately within each population. The prediction accuracy was assessed through the variance proportion explained by the PRS, calculated as $R^{2}=1-\frac{SS1}{SS0}$ where SS0 and SS1 denote the sum of squares for residuals in the null and full models, respectively. The null model incorporated age, sex and the top 20 PCs as covariates, while the full model integrated the PRS and all covariates from the null model. As for the four binary traits we analyzed, we used the logistic model and considered area under the curve (AUC) as the evaluation metric. For any two methods A and B, the percentage of relative improvement of A over B is defined as $({metric}_{A}\;-\;{metric}_{B})/{metric}_{B}\;*\;100\%$. We compared the methods for all traits under two scenarios: without and with a validation dataset. For the first scenario, the prediction accuracy was determined using the entire UK Biobank dataset for the given population. For the second scenario, we randomly split the UK Biobank dataset into validation and testing datasets, and take the median prediction accuracy in 100 random splits.

All the data information discussed above are presented in Table 2.

#### 2.4.2 UK Biobank quantitative traits prediction

Here, we evaluated the performance of JointPRS with other existing methods in predicting 22 quantitative traits.

**Fig. 3:**
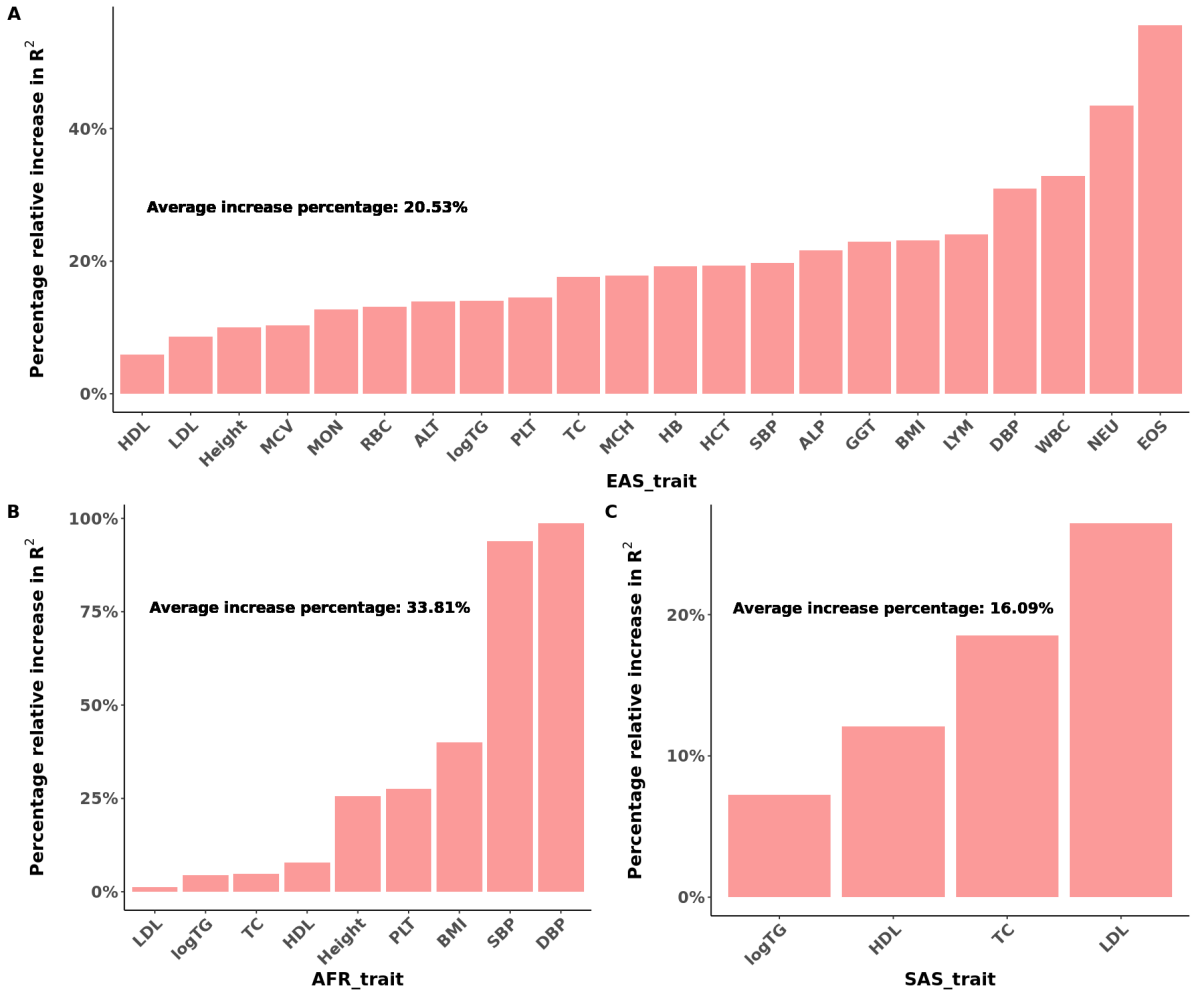
Percentage of PRS Prediction Accuracy Improvement of Using Genetic Correlation in East Asian, African, and South Asian Populations. **A-C**, The relative percentage increase in $R^{2}$ for prediction accuracy of correlation incorporated PRS (JointPRS_auto_2) over PRS-CSx (PRScsx_auto_2) with the auto version using two populations for each method in East Asian (**A**), African (**B**), and South Asian populations (**C**).

We first considered prediction with no validation dataset, and assessed the percentage of prediction accuracy improvement of JointPRS-auto over PRS-CSx-auto across three populations: EAS, AFR, and SAS to show the benefits of considering cross-population genetic correlations. We performed the analysis with the European population as a reference and the respective target populations. As depicted in Figure 3 and Figure S1, the incorporation of the chromosome-wise genetic correlation structure within the joint shrinkage model greatly improved prediction accuracy, with an average of 20.53% improvement for EAS, 33.81% improvement for AFR, and 16.09% improvement for SAS across traits.

**Fig. 4:**
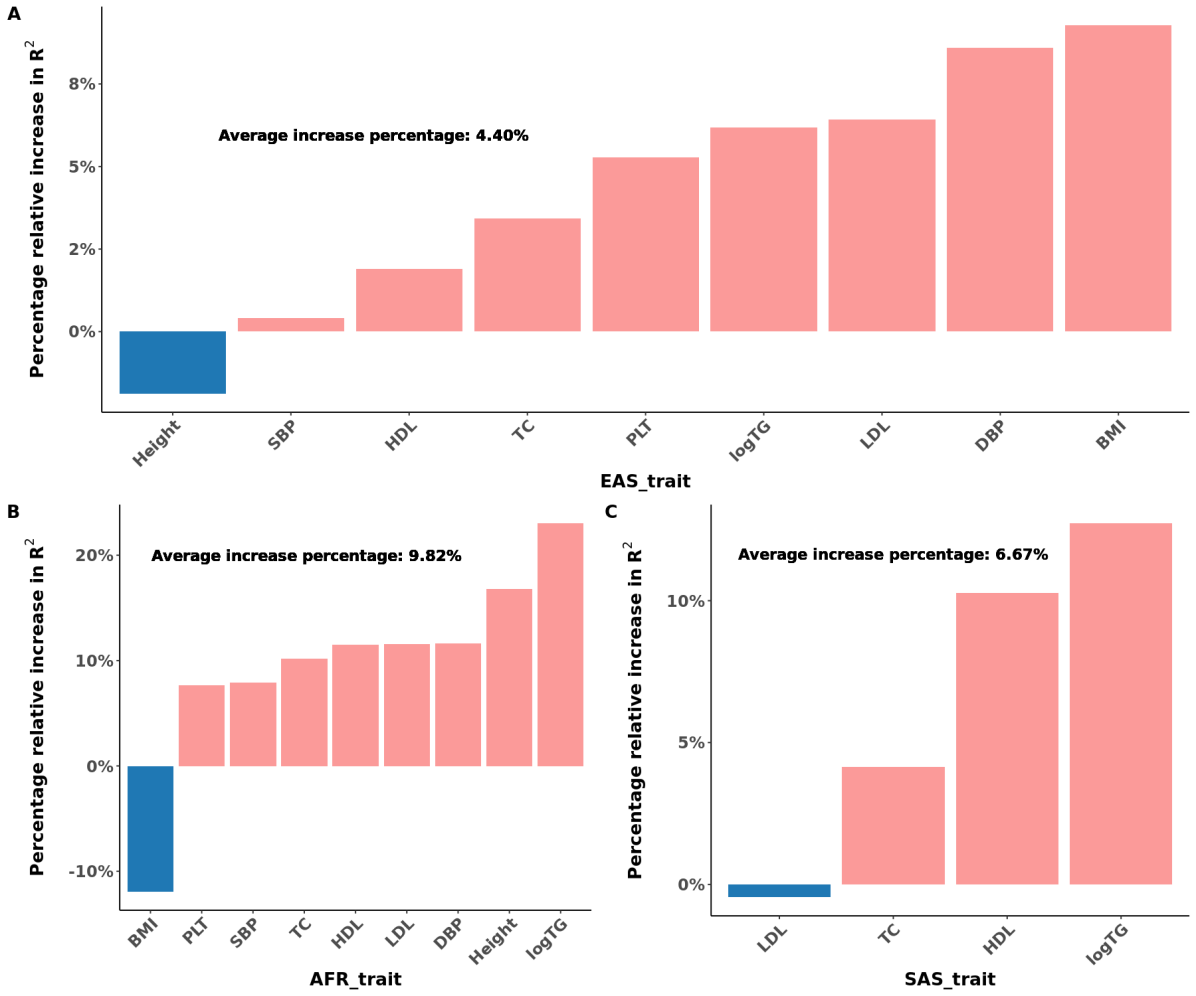
Percentage of PRS Prediction Accuracy Improvement with the Inclusion of Additional Populations in East Asian, African, and South Asian Populations. **A-C**, The relative percentage increase in $R^{2}$ for prediction accuracy of JointPRS with the auto version integrating maximum number of populations available to that trait (JointPRS_auto_max) over JointPRS with the auto version integrating two populations (JointPRS_auto_2) for each method in East Asian (**A**), African (**B**), and South Asian populations (**C**).

Furthermore, for traits with summary statistics from more than two populations, we compared results based on using the maximal number of available populations over results using only two populations. We note the improvement of prediction accuracy with joint consideration of all available populations over using two populations, an average of 4.40% improvement in EAS, 9.82% improvement in AFR, and 6.67% improvement in SAS across traits, as illustrated in Figure 4 and Figure S2.

**Fig. 5:**
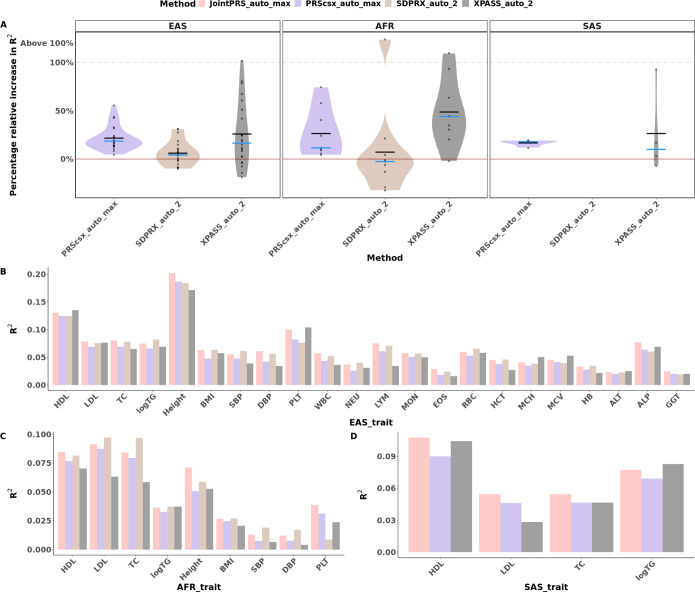
Comparative Analysis of Automated Methods in East Asian, African, and South Asian Populations. **A**, The increased percentage of $R^{2}$ value of JointPRS_auto_max over each method (PRScsx_auto_max,SDPRX_auto_2,XPASS_auto_2) we compared with the auto version using the maximum populations each method can incorporate. Each point represents the increased percentage for each trait, the black crossbar represents the average improvement percentage across traits, and the blue crossbar represents the median of improvement percentage across traits. We are focused on the traits with the improvement of JointPRS over other methods below 100% (including negative improvement), and categorized the traits with improvement above 100% as one group (“Above 100%”) instead of showing the exact value for these traits; **B-D**, The $R^{2}$ value for prediction accuracy of all methods with auto version using the maximum number of populations each method can incorporate for quantitative traits in East Asian (**B**), African (**C**), and South Asian populations (**D**). The evaluation is conducted using all participants that have corresponding phenotypes.

Finally, we compared automatic methods by incorporating the maximum possible number of populations into the analyses: JointPRS_auto_max, PRScsx_auto_max, SDPRX_auto_2, and XPASS_auto_2. As shown in Figure 5 and Table S2, JointPRS-auto performed consistently better than PRS-CSx-auto in all traits in all populations, and had the best performance in 10 out of 22 traits in EAS, 3 out of 9 traits in AFR, and 3 out of 4 traits in SAS. The average relative improvement of JointPRS_auto_max over PRScsx_auto_max measured in R-squared percentage was 21.70% in EAS, 26.40% in AFR, and 16.70% in SAS across traits. Similarly, the average improvement over SDPRX_auto_2 across traits was 6.10% in EAS, and 31.00% in AFR. When compared to XPASS_auto_2, the average improvement of JointPRS_auto_max was 26.60% in EAS, 58.20% in AFR, and 26.40% in SAS across traits. In conclusion, JointPRS had the best performance overall without a validation dataset.

**Fig. 6:**
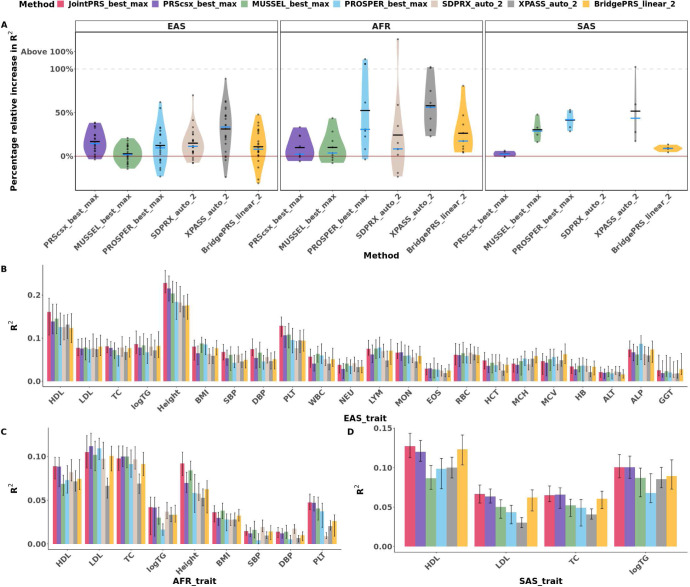
Method Comparisons Using the Optimally-Tuned Parameter in East Asian, African and South Asian Populations. **A**, The median increased percentage of $R^{2}$ value of JointPRS_best_max over each method (PRScsx_best_max, PROSPER_best_max, SDPRX_auto_2, XPASS_auto_2) we compared with the optimally-tuned parameter (or auto) version using the maximum populations each method can incorporate across 100 random splits. Each point represents the median increased percentage for each trait, the black crossbar represents the average for the median improvement percentage across traits, and the blue crossbar represents the median of the median improvement percentage across traits. We are focused on the traits with the improvement of JointPRS over other methods below 100% (including negative improvement), and categorized the traits with improvement above 100% as one group (“Above 100%”) and did not show the exact value for these traits; **B-D**, The $R^{2}$ value for prediction accuracy of all methods with the optimally-tuned parameter (or auto) using the maximum number of populations each method can incorporate for quantitative traits in East Asian (**B**), African (**C**), and South Asian populations (**D**). Selected participants with corresponding phenotypes were randomly split to validation (1/3) and testing dataset (2/3) 100 times. The median, min and max of $R^{2}$ are showed in the barplot **B-D**.

We then compared the methods using the largest number of populations that can be included when there exists a validation dataset. We first compared JointPRS_best_max, PRScsx_best_max, MUSSEL_best_max, PROSPER_best_max, SDPRX_auto_2, XPASS_auto_2, and BridgePRS_linear_2 to evaluate the efficiency of the model using the optimal-tuned parameter(s) for each method without linear combination (or super learning) to guarantee fair comparison. As depicted in Figure 6 and Table S3, JointPRS performed the best in 8 out of 22 traits in EAS, 4 out of 9 traits in AFR, and 2 out of 4 traits in SAS. Specifically, the average relative improvement of JointPRS_best_max over PRScsx_best_max measured in R-squared percentage was 18.80% in EAS, 10.80% in AFR, and 2.61% in SAS across traits. Similarly, the average improvement over MUSSEL_best_max across traits was 5.55% in EAS, 10.60% in AFR, and 31.80% in SAS. And the average improvement over PROSPER_best_max across traits was 15.20% in EAS, 84.10% in AFR, and 43.90% in SAS. Moreover, the average improvement over SDPRX_auto_2 across traits was 14.70% in EAS, and 61.10% in AFR. Compared to XPASS_auto_2, the average improvement was 32.00% in EAS, 61.50% in AFR, and 57.40% in SAS across traits. As for the average improvement over BridgePRS_linear_2 across traits, the improvement was 13.30% in EAS, 29.10% in AFR, and 9.62% in SAS. These results suggest that the JointPRS effectively modeled genetic effect sizes and had the best overall performance when we selected the optimally-tuned parameters for each method.

We further evaluated JointPRS_linear_max, PRScsx_linear_max, MUSSEL_super_max, PROSPER_super_max, SDPRX_linear_2, XPASS_linear_2, and BirdgePRS_linear_2 based on the tuning-parameter strategy outlined in the “existing methods” section, and the results are presented in Figure S3 and Table S4. For this situation, JointPRS_linear_max and PRScsx_linear_max performed similarly, as linear combination diminished the benefits from genetic correlation. When compared to MUSSEL_super_max, the prediction accuracy was similar to JointPRS_linear_max in EAS, and the average relative improvement of JointPRS_linear_max over MUSSEL_super_max measured in R-squared percentage was 6.43% in AFR, and 18.90% in SAS across traits. Similarly, the prediction accuracy of JointPRS_linear_max and PROSPER_super_max was similar to each other in EAS, and the average relative improvement of JointPRS_linear_max over PROSPER_super_max across traits was 31.90% in AFR, and 24.40% in SAS. Moreover, the average improvement over SDPRX_linear_2 across traits was 11.30% in EAS, and 75.60% in AFR. Compared to XPASS_linear_2, the prediction accuracy was similar in EAS, the average improvement across traits was 25.40% in AFR, and 13.40% in SAS. As for the average improvement over BridgePRS_linear_2 across traits, the improvement was 14.00% in EAS, 39.00% in AFR, and 23.90% in SAS. These results suggest that compared to the optimally-tuned parameter model, all methods tend to have more similar performance after linear combination and super learning steps, but JointPRS still had an overall best performance.

In addition, we also compared the prediction accuracy of the tuning version versus the auto version in JointPRS, PRS-CSx, SDPRX and XPASS (JointPRS_linear_max versus JointPRS_auto_max, PRScsx_linear_max versus PRScsx_auto_max, SDPRX_linear_2 versus SDPRX_auto_2, XPASS_linear_2 versus XPASS_auto_2). From Figure S4, we conclude that only JointPRS and SDPRX had similar performance in their tuning version when compared to their corresponding auto version, and JointPRS has better prediction accuracy than SDPRX. This result suggests that JointPRS-auto utilizes pertinent information in the GWAS summary statistics, and the benefits of tuning parameters is limited.

What’s more, we also compared JointPRS_auto_max over other tuning methods including PRScsx_linear_max, PROSPER_super_max, SDPRX_linear_2, XPASS_linear_2, and BridgePRS_linear_2. As illustrated in Figure S5, even when all alternative methods utilized the validation dataset, JointPRS-auto demonstrated performance comparable to these optimally-tuned state-of-the-art methods. This suggests that JointPRS is not only an accurate PRS model but also eliminates the need for tuning parameters.

#### 2.4.3 UK Biobank binary traits prediction

**Fig. 7:**
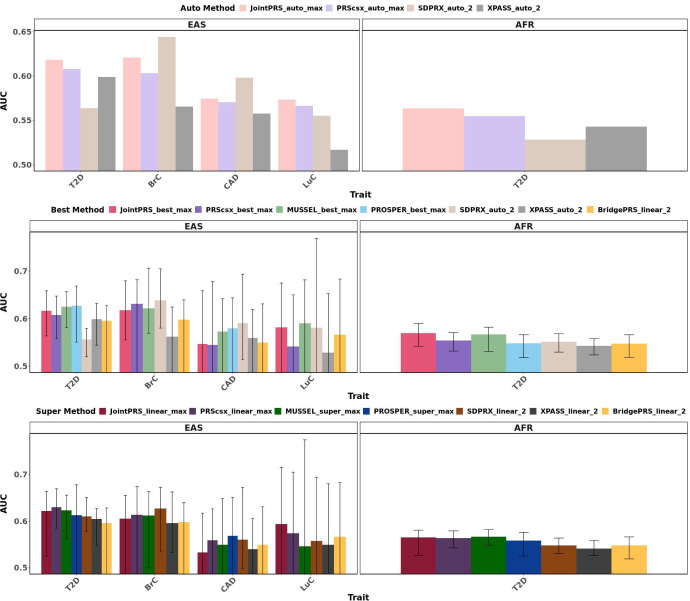
Method Comparisons for Binary Traits in East Asian and African Populations. AUC for methods with auto versions (Auto Method), methods with optimally-tuned parameters (Best Method), and methods with linear combination or super-learning strategies are evaluated for binary traits (Super Method) using the maximum number of populations each method can incorporate. For auto versions, we use the whole dataset for evaluation, while for optimally-tuned parameters or linear combination or super-learning strategies, we randomly split the whole dataset to validation (1/3) and testing dataset (2/3) 100 times, and all tuning steps will be conducted in the validation dataset while all evaluation steps will be conducted in the testing dataset. The median, min and max of $R^{2}$ are showed in the barplot. PROSPER encountered running error in trait BrC and LuC.

We also compared different methods in their prediction of four binary traits based on AUC. Similar to quantitative traits, we considered two scenarios: without and with a validation dataset. The results are summarized in Figure 7 and Table S5, with smaller differences across methods compared to the results with continuous traits. In the absence of a validation dataset, JointPRS-auto performed consistently better than PRS-CSx-auto for all four binary traits in all populations, and performed the best in 2 out of 4 traits in EAS and the only one trait in AFR. The average relative improvement of JointPRS_auto_max over PRScsx_auto_max measured in AUC percentage was 1.63% in EAS, and 1.52% in AFR across traits. Similarly, the average improvement over SDPRX_auto_2 across traits was 1.33% in EAS, and 6.63% in AFR. When compared to XPASS_auto_2, the average improvement across traits was 6.75% in EAS, and 3.78% in AFR. In conclusion, JointPRS outperformed the current leading methods without a validation dataset, but the improvement was more limited compared to quantitative traits result. With a validation dataset, the prediction accuracy measured in AUC was very similar across methods no matter we selected the optimal PRS or combined different PRS using linear combinations or super learning methods.

## 3 Discussion

In this paper, we have introduced JointPRS, an efficient approach only requiring GWAS summary statistics and LD reference data to derive population-specific PRS. Through extensive simulations and real data applications, JointPRS had an overall better performance than the alternative methods, including PRS-CSx [17], PROSPER [19], MUSSEL [18], SDPRX [20], XPASS [21], and BridgePRS [22]. The superior prediction accuracy of JointPRS is attributable to its continuous shrinkage model, the incorporation of a genetic correlation structure, and the simultaneous modeling of multiple populations. Notably, despite introducing additional structures compared to PRS-CSx, JointPRS maintains a comparable computational speed to PRS-CSx (Table S6).

In addition, the JointPRS-auto version also performed well, albeit slightly inferior to the linear combination results. This suggests that the JointPRS model effectively captures most pertinent information, limiting the benefits from learning through the validation dataset. In contrast, there was substantial improvement of PRS-CSx [17] with linear combination, as linear combination implicitly utilized the genetic correlation structure neglected by the plain PRS-CSx model. MUSSEL [18], PROSPER [19], and BridgePRS [22] require parameter fine-tuning due to their setups and thus do not have an auto version. Even though the auto version of XPASS [21] and SDPRX [20] had performances similar to their corresponding linear combination results, their analyses are limited to two populations, restricting the utility of summary statistics and consequently diminishing their performance relative to JointPRS-auto.

There are several advantages for the auto version: acquiring an independent validation dataset is challenging; the efficiency of parameter-tuning highly depends on the sample size of the validation dataset and the degree of similarity between validation and target populations; and the implementation of diverse tuning strategies for application is inconvenient, with methods performance being significantly influenced if not properly tuned. Although various summary statistics-based tuning strategies have been proposed to alleviate these challenges [37–39], further research needs to be conducted to elucidate how to apply these new tuning techniques in state-of-the-art multi-population PRS modeling approaches, as well as their efficacy. Hence, a robust and efficient automatic version of these methods would be highly desirable.

Despite the advantages of JointPRS, we acknowledge several limitations. Firstly, our analysis primarily focused on Hapmap3 SNPs due to the LD reference panels we utilized, consistent with the practice in PRS-CSx, SDPRX, and BridgePRS [17, 20, 22]. However, some studies including MUSSEL and PROSPER [18, 19] suggest that incorporating additional SNPs could enhance prediction accuracy. Moreover, a prediction accuracy disparity still persists between European and non-European populations. This gap cannot be bridged merely with sophisticated statistical models, necessitating an enlarged GWAS sample size for non-European populations [6].

The unified framework of JointPRS can be adapted for the joint modeling of multiple traits, potentially enhancing prediction accuracy as indicated by existing studies [40, 41]. However, such expansion might present challenges, such as the sample overlap issue, necessitating further exploration and investigation.

## 4 Method

In this section, we showed the estimation procedure involving the Markov Chain Monte Carlo (MCMC) and Metropolis-Hastings (MH) algorithms within the JointPRS model.

We first need to obtain the GWAS summary statistics for S SNPS in K populations $\hat{\beta^{1}},\hat{\beta^{2}},\cdots ,\hat{\beta^{K}}$. It is critical to note that some SNP data might be missing in each population, and this issue will be carefully addressed in the subsequent algorithm. In addition, we also need the LD matrix $D^{k}$ for each population k. Due to computational efficiency, the entire genome will be partitioned into $L^{k}$ independent LD blocks based on the reference data for each population k. During each MCMC iteration, SNP effect sizes are updated sequentially for each population k within each LD block $l_{k}$. Then we simplify the notation $l^{k}$ to l during the updates, representing the block variable in the current updating population k. The detailed description of the data preparation and LD block partition is available in the Supplementary Notes (Deta preparation in JointPRS Section).

In the current updating population $k,\;\beta_{\left(l\right)}^{k}={\left(\beta_{\left(l_{1}\right)}^{k},\beta_{\left(l_{2}\right)}^{k},\cdots ,\beta_{\left(l_{s_{l}}\right)}^{k}\right)}^{T}$ represents the effect size vectors for SNPs in block l of population k, with $s_{l}$ indicating the number of SNPs in block l of population k. The marginal effect size estimates for SNPs in block l across K populations are denoted by $\left(\hat{\beta_{\left(l\right)}^{1}},\hat{\beta_{\left(l\right)}^{2}},\cdots ,\hat{\beta_{\left(l\right)}^{K}}\right)$. Additionally, $D_{\left(l\right)}^{k}$ denotes the LD matrix for SNPs in block l of population k, whereas $\Psi_{\left(l\right)}=diag\left(\psi_{\left(l_{1}\right)},\psi_{\left(l_{2}\right)},\cdots ,\psi_{\left(l_{s_{l}}\right)}\right)$ represents the shrinkage matrix for SNPs in block l, and the covariance matrix for SNPs in block l of population k are denoted by $\left(\Sigma_{\left(l_{1}\right)},\Sigma_{\left(l_{2}\right)},\cdots ,\Sigma_{\left(l_{s_{l}}\right)}\right)=\left(\Psi_{l_{1}}M\Sigma M,\Psi_{l_{2}}M\Sigma M,\cdots ,\Psi_{l_{s_{l}}}M\Sigma M\right)$.

As we mentioned before, each SNP j may be unavailable in certain populations, with their missing patterns indicated by $T_{j}$. Then each SNP j can be classified into a group based on its specific missing pattern, represented as $t_{j}$. Consequently, there are up to $2^{K}$ distinct groups in total $1\leq t_{j}\leq 2^{K}$.

### 4.1 MCMC and MH Algorithm in JointPRS

We use 1000×K MCMC iterations with the first 500×K steps as burn-in as suggested by PRS-CSx [17]. For each iteration step, we update parameters by the following procedure:

1. Update $\beta^{1},\beta^{2},\cdots ,\beta^{K}|\left(\hat{\beta^{1}},\hat{\beta^{2}},\cdots ,\hat{\beta^{K}}\right),\;\left(\sigma_{1}^{2},\sigma_{2}^{2},\cdots ,\sigma_{K}^{2}\right),\;D,\;\Psi ,\;\Sigma ,\;M$:
  For the current updating population k in block l, we update the posterior effect size by the following:
(3) $$\begin{array}{l} \beta_{\left(l\right)}^{k}|D_{\left(l\right)}^{k},\hat{\beta_{\left(l\right)}^{k}},\sigma_{k}^{2}\;\sim \;MVN(\mu_{\left(l\right)}^{k},\Sigma_{\left(l\right)}^{k}),\\ \mu_{\left(l\right)}^{k}=\frac{N_{k}}{\sigma_{k}^{2}}\cdot \Sigma_{\left(l\right)}^{k}\cdot \left(\hat{\beta_{\left(l\right)}^{k}}-\frac{\sigma_{k}^{2}}{\sqrt{N_{k}}}\cdot A_{\left(l\right)}^{k}\cdot N_{sqrt}^{k}\right),\\ \Sigma_{\left(l\right)}^{k}=\frac{\sigma_{k}^{2}}{N_{k}}{\left(D_{\left(l\right)}+\sigma_{k}^{2}\cdot diag\left(\frac{{\tilde{\Sigma }}_{kk}^{t_{j}}}{\psi_{\left(l_{j}\right)}}\right)\right)}^{-1}. \end{array}$$
  Here,
$$A_{\left(l\right)}^{k}=\left(\begin{array}{cccccc} \frac{{\tilde{\Sigma }}_{k1}^{t_{1}}}{\psi_{\left(l_{1}\right)}}\beta_{\left(l_{1}\right)}^{1} & \cdots & \frac{{\tilde{\Sigma }}_{kk-1}^{t_{1}}}{\psi_{\left(l_{1}\right)}}\beta_{\left(l_{1}\right)}^{k-1} & \frac{{\tilde{\Sigma }}_{kk+1}^{t_{1}}}{\psi_{\left(l_{1}\right)}}\beta_{\left(l_{1}\right)}^{k+1} & \cdots & \frac{{\tilde{\Sigma }}_{kK}^{t_{1}}}{\psi_{\left(l_{1}\right)}}\beta_{\left(l_{1}\right)}^{K}\\ \vdots & \ddots & \vdots & \vdots & \ddots & \vdots \\ \frac{{\tilde{\Sigma }}_{k1}^{t_{1}}}{\psi_{\left(l_{s_{l}}\right)}}\beta_{\left(l_{s_{l}}\right)}^{1} & \cdots & \frac{{\tilde{\Sigma }}_{kk-1}^{t_{s_{l}}}}{\psi_{\left(l_{s_{l}}\right)}}\beta_{\left(l_{s_{l}}\right)}^{k-1} & \frac{{\tilde{\Sigma }}_{kk+1}^{t_{s_{l}}}}{\psi_{\left(l_{s_{l}}\right)}}\beta_{\left(l_{s_{l}}\right)}^{k+1} & \cdots & \frac{{\tilde{\Sigma }}_{kK}^{t_{s_{l}}}}{\psi_{\left(l_{s_{l}}\right)}}\beta_{\left(l_{s_{l}}\right)}^{K} \end{array}\right),$$
$$N_{sqrt}^{k}={\left(\sqrt{N_{1}}\cdots \sqrt{N_{k-1}}\sqrt{N_{k+1}}\cdots \sqrt{N_{K}}\right)}^{T},$$
$$\tilde{\Sigma }=\left(\begin{array}{cccc} {\tilde{\Sigma }}_{11} & {\tilde{\Sigma }}_{12} & \cdots & {\tilde{\Sigma }}_{1K}\\ {\tilde{\Sigma }}_{21} & {\tilde{\Sigma }}_{22} & \cdots & {\tilde{\Sigma }}_{2K}\\ \vdots & \vdots & \ddots & \ddots \\ {\tilde{\Sigma }}_{K1} & {\tilde{\Sigma }}_{K2} & \cdots & {\tilde{\Sigma }}_{KK} \end{array}\right)=\Sigma^{-1}={\left(\begin{array}{cccc} \sigma_{1}^{2} & \sigma_{12} & \cdots & \sigma_{1K}\\ \sigma_{21} & \sigma_{2}^{2} & \cdots & \sigma_{2K}\\ \vdots & \vdots & \ddots & \vdots \\ \sigma_{K1} & \sigma_{K2} & \cdots & \sigma_{K}^{2} \end{array}\right)}^{-1},$$
  ${\tilde{\Sigma }}^{t_{j}}={\left(\Sigma^{t_{j}}\right)}^{-1}$ the inverse of the covariance matrix for non-missing populations in SNP j.
2. Update $\sigma_{k}^{2}|\Psi ,\;\beta^{k},\;\hat{\beta^{k}},\;D^{k}$:
  For the current updating population k, we update the variance by the following:
(4) $$\sigma_{k}^{2}|\Psi ,\beta^{k},\hat{\beta^{k}},D^{k}\;\sim \;iG\left(\frac{N_{k}\;+\;S_{k}}{2},\frac{N_{k}}{2}\left\{1-2{\sum_{l=1}^{L}\beta_{\left(l\right)}^{k}}^{T}{\hat{\beta }}_{\left(l\right)}^{k}+{\sum_{l=1}^{L}\beta_{\left(l\right)}^{k}}^{T}\left(D_{\left(l\right)}\pm \Psi_{\left(l\right)}^{-1}\right)\beta_{\left(l\right)}^{k}\right\}\right).$$
  Here iG(α,β) is the inverse-gamma distribution with the probability density function
$$f\left(x;\alpha ,\beta \right)=\frac{\beta^{\alpha }}{\Gamma \left(\alpha \right)}{\left(\frac{1}{x}\right)}^{\alpha +1}\exp\left(-\frac{\beta }{x}\right).$$
3. Update each pair of correlation $\rho_{k_{1}k_{2}}$ from the upper triangle under different constraint. Based on the joint model we propose, we can estimate the covariance for two populations $k_{1}$ and $k_{2}$ by assuming:
$$\left(\begin{array}{c} \beta_{j}^{k_{1}}\\ \beta_{j}^{k_{2}} \end{array}\right)\;\sim \;N\left(0,\Psi_{j}M\left(\begin{array}{cc} \sqrt{\sigma_{k_{1}}^{2}} & 0\\ 0 & \sqrt{\sigma_{k_{2}}^{2}} \end{array}\right)\left(\begin{array}{cc} 1 & \rho_{k_{1}k_{2}}\\ \rho_{k_{1}k_{2}} & 1 \end{array}\right)\left(\begin{array}{cc} \sqrt{\sigma_{k_{1}}^{2}} & 0\\ 0 & \sqrt{\sigma_{k_{2}}^{2}} \end{array}\right)M\right).$$
  We note that when we update $\rho_{k_{1}k_{2}}$, we only use SNPs shared by populations $k_{1}$ and $k_{2}$. And if we denote the posterior distribution of $\rho_{k_{1}k_{2}}$ as $h_{r}\left(\rho_{k_{1}k_{2}}\right)$, we have
$$\begin{array}{l} h_{r}\left(\rho_{k_{1}k_{2}}\right)\equiv f\left(\beta |\rho_{k_{1}k_{2}}\right)\\ \;\propto {\left(\sigma_{k_{1}}^{2}\sigma_{k_{2}}^{2}(1-\rho_{k_{1}k_{2}}^{2})\right)}^{-\frac{S_{k_{1}k_{2}}}{2}}exp\left\{-\frac{1}{2}\cdot \frac{N_{k_{1}}N_{k2}}{\sigma_{k_{1}}^{2}\sigma_{k_{2}}^{2}(1\;-\;\rho_{k_{1}k_{2}}^{2})}\cdot \sum_{j=1}^{S_{k_{1}k_{2}}}\;\;\frac{1}{\Psi_{j}}\left(\frac{\sigma_{k_{2}}^{2}}{N_{k_{2}}}\beta_{k_{1}}^{2}-2\frac{\sqrt{\sigma_{k_{1}}^{2}\sigma_{k_{2}}^{2}}\;\cdot \;\rho_{k_{1}k_{2}}}{\sqrt{N_{k_{1}}N_{k_{2}}}}\beta_{j}^{k_{1}}\beta_{j}^{k_{2}}+\frac{\sigma_{k_{1}}^{2}}{N_{k_{1}}}\beta_{k_{2}}^{2}\right)\right\} \end{array}.$$
  Since there is no closed-form distribution to update the correlation $\rho_{k_{1}k_{2}}$, we use the following MH algorithm:

**Algorithm 1:** MH Algorithm for JointPRS

**Ensure:** $\delta_{r}=0.05$	
1:	**while** itr ≤ n_iter **do**
2:	**while** $1\leq k_{1}\leq K-1$ **do**
3:	**while** $k_{1}<k_{2}\leq K$ **do**
4:	$\rho_{k_{1}k_{2}}^{*}=Uniform\left(\rho_{k_{1}k_{2}}-\delta_{r},\rho_{k_{1}k_{2}}+\delta_{r}\right)$
5:	**if** $\rho_{k_{1}k_{2}}^{*}\in [0,cons]$ **then**
6:	$log\_ratio=log\left(h_{r}\left(\rho_{k_{1}k_{2}}^{*}\right)\right)-log\left(h_{r}\left(\rho_{k_{1}k_{2}}\right)\right)$
7:	**if** exp(log_ratio) ≥ *random.Uniform*(0, 1) **then**
8:	$\rho_{k_{1}k_{2}}=\rho_{k_{1}k_{2}}^{*}$
9:	**end if**
10:	**end if**
11:	**end while**
12:	**end while**
13:	**end while**

Note: Here we choose cons = 0 when ϕ = 1E – 6 which is equivalent to the PRS-CSx method to avoid convergence issue and choose cons = 0.99 for all other situations to consider the possible positive correlations between population $k_{1}$ and $k_{2}$.

Then we can also update the corresponding covariance pair $\sigma_{k_{1}k_{2}}=\sqrt{\sigma_{k_{1}}^{2}\sigma_{k_{2}}^{2}}\cdot \;\rho_{k_{1}k_{2}}$.

- Update $\Psi_{j}|\beta_{j},\;\sigma^{2},$ $\delta_{j}$:
  For each SNP j, we update the corresponding shrinkage parameter by the following:
(5) $$\Psi_{j}|\beta_{j},M\Sigma M,\delta_{j}\;\sim \;giG\left(a-\frac{K}{2},2\delta_{j},\beta_{j}{\;}^{T}(M\Sigma M{)}^{-1}\beta_{j}\right)\equiv giG\left(a-\frac{K}{2},2\delta_{j},\beta_{j}{\;}^{T}\tilde{\Sigma }\beta_{j}\right)$$
  Here giG(λ,ρ,χ) is the three-parameter generalized inverse Gaussian distribution with the probability density function
$$f(x;\lambda ,\rho ,\chi )=\frac{(\rho /\chi {)}^{\lambda /2}}{2K_{\lambda }\sqrt{\rho \chi }}x^{\lambda -1}e^{-(\rho x+\chi /x)/2},\;x>0,\;\rho >0,\;\chi >0,$$
  where $K_{\lambda }$ is the modified Bessel function of the second kind.
- Update $\delta_{j}|\Psi_{j}$:
  For each SNP j, we update the distribution parameter by the following:
(6) $$\delta_{j}|\Psi_{j}\;\sim \;G\left(a+b,\Psi_{j}+\phi \right).$$
  Here G(c,d) is the Gamma distribution with the probability density function
$$f(x;c,d)=\frac{(d{)}^{c}}{\Gamma \left(c\right)}(x{)}^{c-1}e^{-d\cdot x}.$$

The detailed proof for the above updating procedure can be found in the Supplementary Notes (MCMC and MH algorithm in JointPRS Section).

### 4.2 Overview of Existing Methods

#### MUSSEL.

The MUSSEL method [18] jointly models GWAS summary statistics and LD structures from multiple populations, utilizing the following multivariate spike-and-slab prior with an incorporated genetic correlation structure for the effect size of each SNP j in each block (J).

$$\left(\begin{array}{c} \beta_{1j}^{J}\\ \vdots \\ \beta_{Kj}^{J} \end{array}\right)\;\sim \;N\left(0,\left(\begin{array}{ccc} \delta_{1j} & \cdots & 0\\ \vdots & \ddots & \vdots \\ 0 & \cdots & \delta_{Kj} \end{array}\right)\left(\begin{array}{ccc} h_{1}^{2} & \cdots & \rho_{1k}h_{1}h_{K}\\ \vdots & \ddots & \vdots \\ \rho_{1k}h_{1}h_{K} & \cdots & h_{K}^{2} \end{array}\right)\left(\begin{array}{ccc} \delta_{1j} & \cdots & 0\\ \vdots & \ddots & \vdots \\ 0 & \cdots & \delta_{Kj} \end{array}\right)\right).$$

This method requires a validation dataset due to the presence of two sets of tuning parameters within the model: the causal SNP proportion and heritability in each population $h_{k}^{2},\;p_{k}(k=1,\cdots ,K)$ and the between-population correlation $\rho_{ij}\;(i\neq j,i=1,\cdots ,K,j=1,\cdots ,K)$. Additionally, a super learning step is introduced to further integrate the scores from various populations and tuning parameters.

#### PROSPER.

The PROSPER method [19] integrates GWAS summary statistics and LD structures from multiple populations, leveraging a linear regression with a combination of Lasso and ridge penalties for estimation in order to consider the spraity of genetic effect sizes and the similarity across populations. The objective function to optimize the effect size vectors of SNP i in K populations can be represented by the following equation

$$\sum_{1\leq i\leq M}\;\left(\beta_{i}^{T}\left(R+\delta_{i}I\right)\beta_{i}-2\beta_{i}^{T}r_{i}+2\lambda_{i}{∥\beta_{i}∥}_{1}^{1}\right)+\sum_{1\leq i_{1}<i_{2}\leq M}\;c_{i_{1}i_{2}}{∥\beta_{i_{1}}^{s_{i_{1}i_{2}}}-\beta_{i_{2}}^{s_{i_{1}i_{2}}}∥}_{2}^{2}.$$

This method requires a validation dataset to determine the tuning parameters associated with these penalties $\left(\delta_{i},\lambda_{i},c_{i_{1}i_{2}},i,i_{1},i_{2}=1,c\ldots M\right)$. A further ensemble step is implemented to combine PRS scores generated across different penalty parameters and populations.

#### PRS-CSx.

The PRS-CSx model [17], an extended Bayesian model of the PRS-CS framework [42], integrates GWAS summary statistics and LD structures from multiple populations by utilizing a shared continuous shrinkage prior for each SNP j in population k as the following equation.

$$\beta_{jk}\;\sim \;N\left(0,\frac{\sigma_{k}^{2}}{N_{k}}\psi_{j}\right),\psi_{j}\;\sim \;\mathrm{Gamma}\left(\delta_{j}\right),\delta_{j}\;\sim \;\mathrm{Gamma}\left(b,\phi \right).$$

In scenarios when a validation dataset is unavailable, the model leverages a full Bayesian approach to estimate the global shrinkage parameter $\phi^{0.5}\;\sim \;C^{+}(0,\;1)$. However, when a validation dataset is available, PRS-CSx evaluates a predefined set of global shrinkage parameters ϕ∈{1E-6,1E-4,1E-2,1,auto}. For each parameter in the set, the obtained scores for each population will be linearly combined to obtain the final score for each population. This integration relies on the validation dataset, and the shrinkage parameter that leads to the best performed combined scores will then be selected based on the prediction accuracy of the validation dataset.

#### SDPRX.

The SDPRX method [20] establishes a hierarchical Bayesian framework, jointly modeling GWAS summary statistics and LD structures from two populations. This framework comprises four components to characterize the genetic architecture, identifying SNPs as no effect, being population-specific, or being shared across two populations. The essential component of this method is the shared component, which uses a mixture of bivariate Gaussian distributions, coupled with a precalculated genetic correlation to approximate the true shared structure. The following equation represent the prior on the effect sizes for each SNP j for population 1 and population 2.

$$\begin{array}{l} \left(\begin{array}{c} \beta_{j1}\\ \beta_{j2} \end{array}\right)\;\;\sim \;p_{0}\left(\begin{array}{c} \delta_{0}\\ \delta_{0} \end{array}\right)+p_{1}\sum_{k=1}^{1000}\;\;\pi_{1k}\left(\begin{array}{c} N\left(0,\delta_{1k}^{2}\right)\\ \delta_{0} \end{array}\right)+p_{2}\sum_{k=1}^{1000}\;\;\pi_{2k}\left(\begin{array}{c} \delta_{0}\\ N\left(0,\delta_{2k}^{2}\right) \end{array}\right)+\\ \;p_{3}\sum_{k=1}^{1000}\;\;\pi_{3k}N\left(\left(\begin{array}{c} 0\\ 0 \end{array}\right),\sigma_{3k}^{2}\left(\begin{array}{cc} 1 & \rho \\ \rho & 1 \end{array}\right)\right). \end{array}$$

Although this method does not need tuning parameters, in the presence of a validation dataset, a linear combination of scores from both populations will be performed to obtain the final score for the target population in our analysis.

#### XPASS.

The XPASS method [21] jointly integrates GWAS summary statistics and LD structures from two populations through a bivariate Gaussian distribution. The genetic correlation structure is incorporated into the model to facilitate the information transference from the auxiliary to the target population. The following model illustrate its idea for the prior on the effect size of each SNP j for population 1 and population 2.

$$\left(\begin{array}{l} \beta_{j1}\\ \beta_{j2} \end{array}\right)\;\sim \;N\left(\left(\begin{array}{l} 0\\ 0 \end{array}\right),\left(\begin{array}{cc} \sigma_{1}^{2} & \rho \sigma_{1}\sigma_{2}\\ \rho \sigma_{1}\sigma_{2} & \sigma_{2}^{2} \end{array}\right)\right).$$

This method is further augmented by considering population-specific effects using selected SNPs based on the P+T procedure, treating them as fixed effects during the estimation. Although this method does not need tuning parameters, when a validation dataset is available, a linear combination of scores from both populations will be performed to derive the final score for the target population in our analysis.

#### BridgePRS.

The BridgePRS method [22] integrates GWAS summary statistics from two populations to consider shared and population-specific SNP effects for the target population. In the first stage, it models the SNP effect sizes for each population under the following zero-centered Gaussian prior

β~N(0,ψλI).

In the second stage, it utilizes the auxiliary population (population 1) to determine the prior of the target population under the following Gaussian model

$$\beta_{2}\;\sim \;N({\tilde{\beta }}_{1},\psi \tau \Omega_{1}).$$

And they finally linearly combined the PRS from the target population with the above joint PRS based on the ridge regression fit.

## Figures and Tables

**Table 1:** Multi-population PRS Methods Summary.

Method type	Method	No validation dataset	Exist a validation dataset	

		auto (automated version)	best (optimal parameter)	linear or super (linear combination or super leaning)

Multiple population model	JointPRS	JointPRS _auto_max	JointPRS_best_max	JointPRS_linear_max
	PRS-CSx [17]	PRScsx_auto_max	PRScsx_best_max	PRScsx_linear_max
	MUSSEL [18]	N/A	MUSSEL_best_max	MUSSEL_super_max
	PROSPER [19]	N/A	PROSPER_best_max	PROSPER_super_max

Two population model	SDPRX [20]	SDPRX_auto_2	SDPRX_auto_2	SDPRX_linear_2
	XPASS [21]	XPASS_auto_2	XPASS_auto_2	XPASS_linear-2
	BridgePRS [22]	N/A	BridgePRS_linear_2	BridgePRS_linear _2

**Table 2:** GWAS Summary Statistics and UK Biobank Data Information.

**Quantitative traits**														

Abbr	Trait	GWAS sample size				GWAS SNP number				UK Biobank sample size				UK Biobank Field

		EUR	EAS	AFR	SAS	EUR	EAS	AFR	SAS	EUR	EAS	AFR	SAS	

HDL	HDL-cholesterol	885,546 [8]	116,404 [8]	90,804 [8]	33,953 [8]	800,281	735,249	827,727	1,085,452	271,876	1,812	5,927	6,784	30760
LDL	LDL-cholesterol	840,012 [8]	79,693 [8]	87,759 [8]	33,658 [8]	800,283	797,861	827,727	1,088,264	294,412	1,911	6,171	7,062	30780
TC	Total cholesterol	929,739 [8]	144,579 [8]	92,554 [8]	34,135 [8]	800,281	461,893	827,727	1,085,270	294,966	1,912	6,183	7,080	30690
logTG	Triglycerides	860,679 [8]	81,071 [8]	89,467 [8]	34,023 [8]	800,286	797,898	827,727	1,088,215	296,706	1,993	6,400	7,444	30870
Height	Height	252,357 [26]	159,095 [9]	49,781 [10]	N/A	724,431	790,675	827,738	N/A	310,797	2,080	6,574	N/A	50
BMI	Body mass index	233,787 [27]	158,284 [11]	49,335 [10]	N/A	725,221	782,322	827,738	N/A	310,455	2,077	6,713	N/A	21001
SBP	Systolic blood pressure	728,893 [28]	179,000 [12]	35,433 [10]	N/A	797,661	747,306	827,738	N/A	308,802	2,001	6,688	N/A	93; 4080
DBP	Diastolic blood pressure	746,038 [28]	179,000 [12]	35,433 [10]	N/A	798,292	747,306	827,738	N/A	308,808	2,001	6,574	N/A	94; 4090
PLT	Platelet	539,667 [29]	179,000 [12]	29,328 [10]	N/A	800,321	747,306	827,738	N/A	302,170	2,027	6,445	N/A	30080
WBC	White blood cell	559,083 [29]	179,000 [12]	N/A	N/A	800,320	747,306	N/A	N/A	302,166	2,027	N/A	N/A	30000
NEU	Neutrophil	517,889 [29]	179,000 [12]	N/A	N/A	800,319	747,306	N/A	N/A	301,618	2,025	N/A	N/A	30140
LYM	Lymphocyte	523,524 [29]	179,000 [12]	N/A	N/A	800,320	747,306	N/A	N/A	301,618	2,025	N/A	N/A	30120
MON	Monocyte	520,195 [29]	179,000 [12]	N/A	N/A	800,320	747,306	N/A	N/A	301,618	2,025	N/A	N/A	30130
EOS	Eosinophil	473,152 [29]	179,000 [12]	N/A	N/A	800,319	747,306	N/A	N/A	301,618	2,025	N/A	N/A	30150
RBC	Red blood cell	542,043 [29]	179,000 [12]	N/A	N/A	800,318	747,306	N/A	N/A	302,170	2,027	N/A	N/A	30010
HCT	Hematocrit	559,099 [29]	179,000 [12]	N/A	N/A	800,319	747,306	N/A	N/A	302,170	2,027	N/A	N/A	30030
MCH	Mean corpuscular hemoglobin	483,664 [29]	179,000 [12]	N/A	N/A	800,319	747,306	N/A	N/A	302,167	2,027	N/A	N/A	30050
MCV	Mean corpuscular volume	540,967 [29]	179,000 [12]	N/A	N/A	800,317	747,306	N/A	N/A	302,170	2,027	N/A	N/A	30040
HB	Hemoglobin	408,112 [29]	179,000 [12]	N/A	N/A	800,322	747,306	N/A	N/A	302,170	2,027	N/A	N/A	30020
ALT	Alanine aminotransferase	437,267 [30]	179,000 [12]	N/A	N/A	787,866	747,306	N/A	N/A	296,840	1,993	N/A	N/A	30620
ALP	Alkaline phosphatase	437,267 [30]	179,000 [12]	N/A	N/A	799,800	747,306	N/A	N/A	296,959	1,994	N/A	N/A	30610
GGT	γ-glutamyl transpeptidase	437,267 [30]	179,000 [12]	N/A	N/A	799,800	747,306	N/A	N/A	296,796	1,991	N/A	N/A	30730

Binary traits																

Abbr	Trait	GWAS sample size						GWAS SNP number			UK Biobank sample size					

		EUR		EAS		AFR					EUR		EAS		AFR	
		case	control	case	control	case	control	EUR	EAS	AFR	case	control	case	control	case	control

T2D	Type 2 diabetes	26,676	132,532 [31]	36,614	155,150 [14]	14,042	31,683 [10]	800,320	799,544	827,738	25,309	286,197	203	1,887	1,292	5,535
BrC	Breast cancer	46,785	42,892 [32]	5,552	89,731 [15]	N/A	N/A	799,126	786,215	N/A	13,973	297,533	89	2,001	N/A	N/A
CAD	Coronary artery disease	22,233	64,762 [33]	29,319	183,134 [15]	N/A	N/A	653,825	786,215	N/A	23,271	288,235	56	2,034	N/A	N/A
LuC	Lung cancer	29,266	56,450 [34]	4,050	208,403 [15]	N/A	N/A	756,305	786,215	N/A	4,107	307,399	21	2,069	N/A	N/A

## Data Availability

BBJ summary statistics, http://jenger.riken.jp/en/result, https://humandbs.biosciencedbc.jp/en/hum0197-v3-220 BCAC summary statistics, https://www.ebi.ac.uk/gwas/publications/25751625 BCX summary statistics, http://www.mhi-humangenetics.org/en/resources/ CARDIoGRAM summary statistics, https://www.ebi.ac.uk/gwas/publications/21378990 TRICL-ILCCO and LC3, https://www.ebi.ac.uk/gwas/publications/28604730 DIAGRAM summary statistics, https://www.ebi.ac.uk/gwas/publications/28566273 GIANT summary statistics, https://portals.broadinstitute.org/collaboration/giant/index.php/GIANT_consortium_data_files GLGC summary statistics, https://csg.sph.umich.edu/willer/public/glgc-lipids2021/ ICBP summary statistics, https://www.ebi.ac.uk/gwas/publications/30224653 PAGE summary statistics, https://www.ebi.ac.uk/gwas/publications/31217584 UKB summary statistics, https://www.ebi.ac.uk/gwas/publications/33972514
